# Long-range allosteric communication within antibodies affects antigen-binding affinity

**DOI:** 10.3389/fimmu.2026.1865402

**Published:** 2026-07-03

**Authors:** Susan K. Vester, Brian J. Sutton, James M. McDonnell

**Affiliations:** Randall Centre for Cell and Molecular Biophysics, King’s College London, New Hunt’s House, London, United Kingdom

**Keywords:** affinity, allostery, antibody, antigen, Fab, Fc, isotype, thermodynamics

## Abstract

As an essential component of the immune system, antibodies are among the most rigorously studied molecules in the biosciences; they combine antigen recognition with isotype-dependent effector functions. Despite this, allosteric communication within antibodies and the resulting effects on antigen-binding affinities, specifically those conferred by antibody class (isotype) or subclass, remain poorly understood. Using surface plasmon resonance, we performed a comprehensive comparison of IgA_1_, IgD, IgE, IgG_1_, IgG_4_ and IgM Fabs across five distinct antibody-antigen systems. While different C_H_1 domains produced small, medium-range allosteric differences in affinity, we identified much larger, long-range effects transmitted from the Fc region to the Fab region. Full-length antibodies consistently exhibited higher binding affinities than their Fab counterparts, independent of avidity. Thermodynamic analysis indicates these effects are driven by differences in antibody flexibility and pre-organization. Taken together, these findings demonstrate that allosteric modulation of antigen binding - mediated by the C_H_1 domain, the Fc region, and Fc-ligand interactions - is a critical determinant of antibody function, offering essential insights for the assessment and engineering of diverse therapeutic formats.

## Introduction

Antibodies are an important component of the adaptive immune system, produced in response to microbial pathogens. In humans, there are nine different antibody classes and subclasses (IgA_1_, IgA_2_, IgD, IgE, IgG_1_, IgG_2_, IgG_3_, IgG_4_ and IgM), which differ in their heavy chain constant domains and glycosylation patterns, and consequently bind to different receptors, mediate different effector functions, have different half-lives and can be found in different oligomerization states (1). Antibodies are made up of two heavy and two light chains, with the antigen-binding site, or paratope, formed by parts of the variable domains of heavy (V_H_) and light chain (V_L_) (2). Antibodies combine the properties of antigen recognition through their variable (V) domains within the two Fab regions, with isotype-dependent effector functions mediated by their heavy chain constant domains within the Fc region; for many years these functions were thought to be independent. In the IgA, IgD and IgG isotypes, the Fab and Fc regions are connected by a hinge region, while IgE and IgM have an additional constant heavy chain (C_H_) domain but lack a hinge (3). An increasing body of evidence suggests that antigen binding is not completely independent of antibody isotype, with allosteric effects between V and constant (C) domains modulating antigen binding (4). One of the first studies, comparing the binding kinetics and affinities of mouse IgG subclasses using surface plasmon resonance (SPR), reported small differences in association rates, dissociation rates and affinity values in binding to antigen, and noted the impact of antigen density on their results (5). Unless a very thorough analysis is carried out, binding of bivalent antibodies to their immobilized antigens may be affected by avidity effects; the antibody’s molecular reach, oligomerization state and antigen density can affect the affinity measurements, and care must be taken when comparing binding kinetics and affinities of different antibody isotypes in this way. Some studies have reported small differences in antigen-binding affinities between subsets of human or mouse antibody isotypes using isothermal titration calorimetry (ITC) or SPR (6–8). One comprehensive analysis compared the binding of therapeutic Trastuzumab and Pertuzumab (produced as each of the nine human antibody classes and subclasses) to HER2 using biolayer interferometry; when binding of antigen to the different antibody isotypes was assessed independent of valency and avidity effects, only subtle differences in binding kinetics and affinities between the isotypes were found (9). Other studies have reported larger differences in affinity between IgA and IgG Fabs, suggesting that the C_H_1 domain can substantially influence binding kinetics and affinities (10, 11). However, careful biophysical characterization is not always performed in studies comparing the binding affinity of different antibody isotypes and reported effect sizes vary widely. While it is more intuitive to understand how the C_H_1 domain might affect antigen binding in a medium-range allosteric manner through subtle modifications to the V_H_-V_L_ interface (12), there have also been reports of long-range allosteric effects, such as mutations in the Fc region of IgA altering antigen binding (13). Correspondingly, long-range allosteric effects on the binding affinity of antibody receptors have been reported in the presence or absence of antigen, such as differences in the binding of IgG to the neonatal Fc receptor (14).

We report here a thorough biophysical comparison of all five human antibody classes, including two IgG subclasses, for five different antibody-antigen interactions, initially focusing on Fabs and the effects on antigen binding conferred by the different C_H_1 domains. We find that differences in antigen affinity between antibody isotypes are less than two-fold in magnitude. However, we observed much larger, long-range allosteric effects on antigen binding, caused by the presence of the Fc region, or when, for IgE, different antibody capture methods for the IgE-Fc were used. Thermodynamic analysis reveals that these Fab-Fc long-range allosteric effects appeared to be mediated by antibody flexibility.

## Materials and methods

### Plasmids and cloning

Cloning was performed using standard PCR methods with Q5 High-Fidelity 2X Master Mix and NEBuilder HiFi DNA Assembly Cloning Kit (both New England Biolabs). All protein-coding sequences were verified by Sanger sequencing (Source Bioscience or Eurofins Genomics). The constructs for pVITRO1-human anti-Phl p 7 Ig 102.1F10 (HAPPI1) Fabs and full-length antibodies with a C-terminal glycine_4_-serine linker and His_6_-tag on the heavy chain were derived from the respective pVITRO1 full-length constructs of IgA_1_ (Addgene 50370) (15), IgD (Addgene 204626) (16), IgE (Addgene 50365) (15), IgG_1_ (Addgene 50366) (15), IgG_4_ (Addgene 50369) (15) or IgM (Addgene 61880) (15). The pVITRO1-human anti-Phl p 7 Ig CS09G6K (HAPPI2) (17) Fab constructs were derived from pVITRO1-HAPPID2 Fab (16) and the above His-tagged pVITRO1-HAPPI1 Fabs. The V_H_ and V_L_ domains of the human anti-Zika virus envelope glycoprotein domain III antibody ZK2B10 (18, 19) were codon-optimized for human expression, synthetized by GenScript, and cloned into His-tagged pVITRO1-HAPPI1 Fab constructs giving pVITRO1-human anti-Zika virus envelope glycoprotein domain III antibody ZK2B10 (anti-EDIII) Fabs. The constructs for pVITRO1-human anti-*Plasmodium falciparum* circumsporozoite protein (PfCSP) antibody CIS43 (anti-PfCSP) Fabs were derived from pVITRO1-CIS43 IgE (20) and His-tagged pVITRO1-HAPPI2 Fabs. The V_H_ and V_L_ domains of human anti-HIV-1 gp120 variable loop 3 (V3) antibody TA6 (21, 22) were codon-optimized for human expression, synthetized by GenScript, and cloned into His-tagged pVITRO1-HAPPI1 Fab constructs giving pVITRO1-human anti-HIV-1 gp120 V3 antibody TA6 (anti-gp120) Fabs. Sequence alignments were performed with Clustal Omega version 1.2.4 (23) (**Supplementary Figure 1**).

The constructs for pET151-Phl p 7 with a C-terminal tryptophan residue (24) and pET-15b-anti-IgD nanobody 072 (aδNb072) (Addgene 204627) (16) have been described. The pET-28a construct for Zika virus envelope glycoprotein domain III (ZIKV-EDIII) (19) was synthetized by GenScript, codon-optimized for *E. coli* expression. The pET-28a-MBP-super TEV protease plasmid (Addgene 171782) was a gift from Mark Howarth and used for expression and purification of MBP-super TEV protease (25).

### Antibody expression and purification

Antibody Fabs were transiently expressed in Expi293F cells, maintained in Expi293 Expression Medium, using the ExpiFectamine 293 Transfection Kit (all Thermo Fisher) according to the manufacturer’s instructions. Full-length antibodies were expressed from stably transfected FreeStyle 293-F cells (Thermo Fisher), prepared by hygromycin selection, in FreeStyle 293 Expression Medium supplemented with 50 µg/mL hygromycin B (both Thermo Fisher) at 37 °C with 5% CO_2_ in spinner flasks. Supernatants were harvested by centrifugation and filtered through 0.2 µm PES. Fabs and full-length antibodies were purified by Ni-NTA Superflow resin (Qiagen) and desalted into HBS-az (10 mM HEPES, 150 mM NaCl, pH 7.4 + 0.1% NaN_3_). HAPPI1 Fabs and full-length antibodies were further purified by size exclusion chromatography (SEC) on a Superdex 200 Increase 10/300 GL column (Cytiva) in HBS-az. Human HAPPIE1 without a His_6_-tag was expressed from a stably transfected HEK293F cell line and purified by anti-IgE affinity chromatography and SEC as previously described (24).

### Antigen expression and purification

Phl p 7 was expressed in BL21(DE3) *E. coli* (New England Biolabs) in ZYP-5052 autoinduction medium (26) at 30 °C, purified using Ni-NTA Superflow resin and desalted into HBS-Ca-az (10 mM HEPES, 150 mM NaCl, 4 mM CaCl_2_, pH 7.4 + 0.1% NaN_3_). The N-terminal His_6_-tag was removed by incubation with MBP-super TEV protease at a 20:1 molar ratio overnight at 4 °C, with remaining His-tagged protein removed by incubation with HIS-Select Nickel Affinity Gel (Merck). Phl p 7 was further purified by SEC on a Superdex 75 Increase 10/300 GL column (Cytiva) in HBS-Ca-az.

ZIKV-EDIII was expressed in BL21(DE3) cells in ZYP-5052 autoinduction medium at 37 °C and refolded from inclusion bodies, similarly to what has previously been described (27). In brief, cells were lysed by incubation in BugBuster Protein Extraction Reagent supplemented with lysonase (both Merck) at room temperature (RT) for 30 min under constant agitation. Inclusion bodies were pelleted at 10015 × *g* for 15 min at 4 °C, then washed twice with 10 mM Tris pH 8.6 (at 4 °C) + 0.1 mM EDTA + 500 mM LiCl + 0.5% (v/v) Nonidet P40 Substitute + 1 mM DTT and twice washed in the same buffer containing no LiCl. Inclusion bodies were solubilized by incubation in 6 M guanidine hydrochloride + 10 mM DTT at RT for 30 min, then centrifuged at 16000 × *g* for 5 min. ZIKV-EDIII was refolded in 100 mM Tris pH 8.6 (at 4 °C) + 400 mM L-arginine + 2 mM EDTA + 0.2 mM PMSF + 5 mM reduced glutathione + 0.5 mM oxidized glutathione at a 1:100 dilution overnight at 4 °C. The refold was desalted into HBS-az, concentrated, and purified by SEC using a Superdex 75 Increase 10/300 GL column in HBS-az. The N-terminal His_6_-tag was removed by incubation with MBP-super TEV protease at a 20:1 molar ratio at 4 °C overnight, with remaining His-tagged protein removed as above.

PfCSP peptide 21 (NPDPNANPNVDPNAN) and biotinylated HIV-1 gp120 V3 peptide from the NY5 isolate (NNTKKGIAIGPGRTLYAREK) were purchased from GenScript.

### Other protein expression and purification

aδNb072 was expressed and purified as described previously (16). The C-terminal His_6_-tag of aδNb072 was removed by incubation with MBP-super TEV protease at a 10:1 molar ratio for 2 hours at 34 °C, with remaining His-tagged protein removed as above. The soluble form of the IgE high-affinity receptor FcϵRI α-chain (sFcϵRIα) was expressed and purified as an IgG_4_-Fc fusion protein as described previously (28). Omalizumab Fab was expressed and purified as previously described (29).

### Surface plasmon resonance

SPR experiments were performed using a Biacore T200 (Cytiva) at 25 °C, unless otherwise specified for thermodynamic analysis, in HBS-P+ (10 mM HEPES + 150 mM NaCl, pH 7.4 + 0.05% surfactant P20). For experiments with the calcium-dependent polcalcin Phl p 7, the running buffer was supplemented with 5 mM CaCl_2_.

Anti-His-tag chips were prepared using the His Capture and Amine Coupling Kits on a CM5 sensor chip (all Cytiva), adapted from the manufacturer’s instructions. His-tagged Fabs were captured at different concentrations for 180 s at 10 µL/min to achieve similar capture levels for the antibody isotypes. 100 nM His-tagged antibodies were captured at 5 µL/min for varying durations to achieve similar capture levels for the antibody isotypes. For thermodynamic experiments, 50 nM His-tagged HAPPIG_1_1 Fab and antibody were captured at 5 µL/min for 240 s. Two-fold dilution series of antigen, aδNb072 or peptide were flowed over for 240 s or 120 s at 20 µL/min, with a dissociation phase of 900 s or 600 s. Regeneration was performed with 0.1 M glycine pH 2.0 for 60 s at 10 µL/min.

A differential IgE pre-capture chip was prepared by covalently immobilizing sFcϵRIα and omalizumab Fab onto a CM5 sensor chip using the Amine Coupling Kit, adapted from the manufacturer’s instructions. 50 nM HAPPIE1 was captured for 180 s at 10 µL/min. A two-fold dilution series of Phl p 7 was flowed over for 240 s at 20 µL/min, with a dissociation phase of 900 s. Regeneration was performed with 0.1 M glycine pH 2.0 for 60 s at 10 µL/min.

All experiments were carried out in duplicate, using the same dilution of antibody and antigen for independent measurements. Double-reference subtraction was performed using Biacore T200 Evaluation software version 1.0 (Cytiva) (30). Data were plotted and fit using Origin 7 (OriginLab Corporation). For kinetic binding analysis, association phase data (including data until a plateau was reached) were used to fit k_obs_ values using the equation y = B_eq_ × (1 – exp(–x×k_obs_)); k_obs_ values were plotted against concentration and k_on_ values were derived from the slope of a linear fit (31). Dissociation phase data were used to derive k_off_ values using the in-built single or double exponential decay function. The ratio of k_off_/k_on_ was used to determine K_D_ values. For equilibrium binding analysis, maximal response at equilibrium (before an artifactual downturn in signal occurred) was plotted against concentration, and K_D_ values were determined using the in-built one site direct binding function. For thermodynamic analysis, k_on_, k_off_ and K_D_ values were derived using kinetic binding analysis as above. K_D_ values were used for van ‘t Hoff analysis to derive the equilibrium ΔH value from the slope of a linear fit using the equation R × ln(1/K_D_) = –ΔH/T + ΔS, with the gas constant R = 8.314 J mol^-1^ K^-1^. ΔG values were derived using the equation ΔG = –R × T × ln(1/K_D_) and used to calculate –TΔS values using the equation –T × ΔS = ΔG – ΔH, as described previously (32). Values for k_on_ and k_off_ were used for transition state analysis using the Eyring equation, with the ΔH^‡^ value derived from the slope of a linear fit using the equation R × ln((k×*h*)/(k_B_×T)) = –ΔH^‡^/T + ΔS^‡^, with the Planck constant *h* = 6.63×10^–34^ J s and Boltzmann constant k_B_ = 1.38×10^–23^ J K^-1^, as previously described (32). ΔG^‡^ was estimated using the equation ΔG^‡^ = –R × T × ln((k×*h*)/(k_B_×T)), and –TΔS^‡^ values derived from –T × ΔS^‡^ = ΔG^‡^ – ΔH^‡^.

## Results

### The C_H_1 domain has a small effect on antigen-binding affinity

Using SPR, we first assessed how the different C_H_1 domains from the antibody isotypes IgA_1_, IgD, IgE, IgG_1_, IgG_4_ and IgM affect antigen-binding affinity and kinetics. We chose five different antibodies, two specific for the same grass pollen allergen, two against different viral envelope glycoproteins, and one directed to a malarial surface protein, and expressed each in Fab format for the six specified isotypes with a His_6_-tag on their heavy chain (**Table 1**; **Supplementary Figures 1**, **2A**). Capturing the Fabs via their His_6_-tag on an anti-His-tag sensor chip, we flowed over a two-fold dilution series of antigen. In this way, the same dilution of antigen was used to measure binding to all Fabs, with three Fabs assayed simultaneously, eliminating concentration variability of analytes.

**Table 1:** Overview of the antibody-antigen systems used in this study.

Name	Antigen	Antibody	V_H_	V_L_	First described	Antigen-bound structure
HAPPI1	Phl p 7 (protein)	102.1F10	IGHV1-3	IGLV1-40	(33)	(17)
HAPPI2	Phl p 7 (protein)	CS09G6K	IGHV4-39	IGKV1-6	(17)	N/A
anti-EDIII	ZIKV-EDIII (protein)	ZK2B10	IGHV1-8	IGLV1-47	(18)	(19)
anti-PfCSP	PfCSP peptide 21	CIS43	IGHV1-3	IGKV4-1	(34)	(34)
anti-gp120	HIV-1 gp120 V3 (peptide)	TA6	IGHV1-3	IGLV3-10	(21)	(22)

HAPPI1, human anti-Phl p 7 Ig 102.1F10; HAPPI2, human anti-Phl p 7 Ig CS09G6K; anti-EDIII, human anti-Zika virus envelope glycoprotein domain III antibody ZK2B10; anti-PfCSP, human anti-*Plasmodium falciparum* circumsporozoite protein antibody CIS43; anti-gp120, human anti-HIV-1 gp120 variable loop 3 antibody TA6; ZIKV-EDIII, Zika virus envelope glycoprotein domain III; PfCSP, *Plasmodium falciparum* circumsporozoite protein; V3, variable loop 3; N/A, not available.

**Figure 1:**
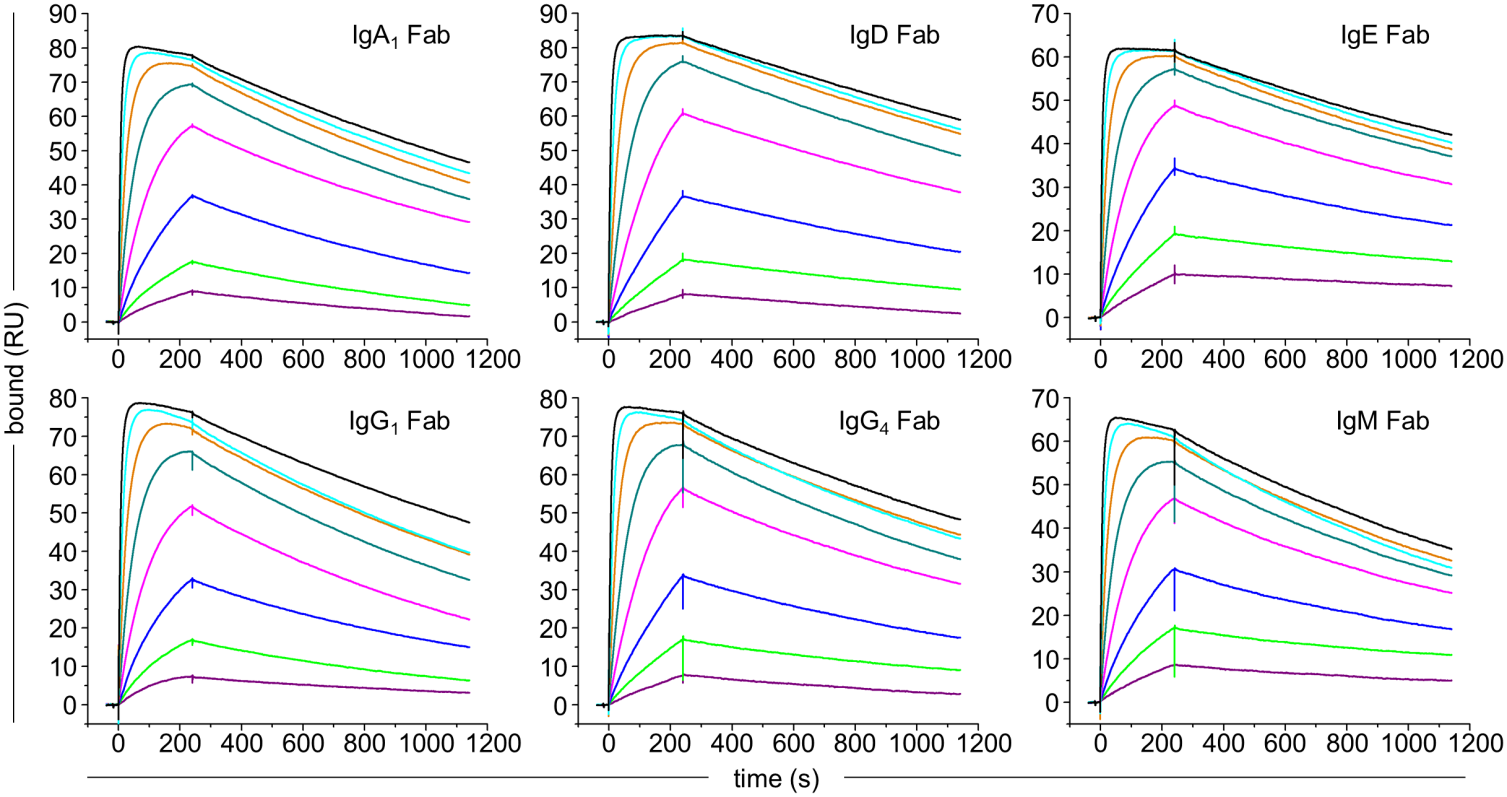
Binding of the antigen Phl p 7 to HAPPI1 Fabs by SPR. HAPPI1 Fabs were captured on an anti-His-tag chip and the grass pollen allergen Phl p 7 was flowed over in a two-fold dilution series, with the highest concentration 100 nM (black line) and the lowest concentration 1 nM (purple line). HAPPI1 Fabs were captured at slightly different densities. RU, resonance units.

First we tested binding of the grass pollen allergen Phl p 7 to the human anti-Phl p 7 IgA_1_ 102.1F10 (HAPPIA_1_1), human anti-Phl p 7 IgD 102.1F10 (HAPPID1), human anti-Phl p 7 IgE 102.1F10 (HAPPIE1), human anti-Phl p 7 IgG_1_ 102.1F10 (HAPPIG_1_1), human anti-Phl p 7 IgG_4_ 102.1F10 (HAPPIG_4_1) and human anti-Phl p 7 IgM 102.1F10 (HAPPIM1) Fabs (**Figure 1**; **Table 2**; **Supplementary Figure 3A**). Binding affinities ranged between 0.26 nM and 0.48 nM, with a ~1.8-fold difference between the highest affinity (IgE Fab) and lowest affinity (IgG_1_ Fab). Small differences were found in both k_on_ and k_off_ values, which ranged up to ~1.4- and ~1.5-fold, respectively.

**Table 2:** Kinetics and affinities of antigens binding to Fabs.

Antigen	Antibody	k_on_ ± SD (M^-1^ s^-1^)	k_off_ ± SD (s^-1^)	K_D_ ± SD (M)
Phl p 7 to	HAPPIA_1_1 Fab	1.6 (± 0.1) × 10^6^	6.5 (± 0.5) × 10^-4^	4.1 (± 0.4) × 10^-10^
	HAPPID1 Fab	1.4 (± 0.1) × 10^6^	4.8 (± 0.1) × 10^-4^	3.4 (± 0.1) × 10^-10^
	HAPPIE1 Fab	1.9 (± 0.1) × 10^6^	4.8 (± 0.1) × 10^-4^	2.6 (± 0.1) × 10^-10^
	HAPPIG_1_1 Fab	1.5 (± 0.1) × 10^6^	7.0 (± 0.4) × 10^-4^	4.8 (± 0.3) × 10^-10^
	HAPPIG_4_1 Fab	1.5 (± 0.1) × 10^6^	5.9 (± 0.2) × 10^-4^	4.0 (± 0.1) × 10^-10^
	HAPPIM1 Fab	1.6 (± 0.1) × 10^6^	7.3 (± 0.4) × 10^-4^	4.4 (± 0.3) × 10^-10^
Phl p 7 to	HAPPIA_1_2 Fab	9.4 (± 0.1) × 10^5^	8.1 (± 0.3) × 10^-4^	0.9 (± 0.1) × 10^-9^
	HAPPID2 Fab	7.8 (± 0.1) × 10^5^	8.3 (± 0.2) × 10^-4^	1.1 (± 0.1) × 10^-9^
	HAPPIE2 Fab	9.2 (± 0.1) × 10^5^	7.4 (± 0.4) × 10^-4^	0.8 (± 0.1) × 10^-9^
	HAPPIG_1_2 Fab	7.1 (± 0.1) × 10^5^	9.0 (± 0.1) × 10^-4^	1.3 (± 0.1) × 10^-9^
	HAPPIG_4_2 Fab	8.4 (± 0.1) × 10^5^	7.6 (± 0.1) × 10^-4^	0.9 (± 0.1) × 10^-9^
	HAPPIM2 Fab	8.4 (± 0.1) × 10^5^	9.7 (± 0.2) × 10^-4^	1.1 (± 0.1) × 10^-9^
ZIKV-EDIII to	anti-EDIII IgA_1_ Fab	2.3 (± 0.1) × 10^5^	1.4 (± 0.1) × 10^-3^	5.9 (± 0.1) × 10^-9^
	anti-EDIII IgD Fab	2.3 (± 0.1) × 10^5^	1.2 (± 0.1) × 10^-3^	5.2 (± 0.1) × 10^-9^
	anti-EDIII IgE Fab	2.5 (± 0.1) × 10^5^	1.3 (± 0.1) × 10^-3^	5.0 (± 0.1) × 10^-9^
	anti-EDIII IgG_1_ Fab	2.2 (± 0.1) × 10^5^	1.4 (± 0.1) × 10^-3^	6.3 (± 0.1) × 10^-9^
	anti-EDIII IgG_4_ Fab	2.5 (± 0.1) × 10^5^	1.3 (± 0.1) × 10^-3^	5.2 (± 0.1) × 10^-9^
	anti-EDIII IgM Fab	2.3 (± 0.1) × 10^5^	1.6 (± 0.1) × 10^-3^	6.9 (± 0.1) × 10^-9^
peptide 21 to	anti-PfCSP IgA_1_ Fab	7.2 (± 0.1) × 10^5^	4.5 (± 0.1) × 10^-3^	6.2 (± 0.1) × 10^-9^
	anti-PfCSP IgD Fab	5.0 (± 0.2) × 10^5^	3.5 (± 0.1) × 10^-3^	6.9 (± 0.3) × 10^-9^
	anti-PfCSP IgE Fab	8.1 (± 0.2) × 10^5^	4.3 (± 0.1) × 10^-3^	5.3 (± 0.3) × 10^-9^
	anti-PfCSP IgG_1_ Fab	6.6 (± 0.1) × 10^5^	4.2 (± 0.2) × 10^-3^	6.4 (± 0.2) × 10^-9^
	anti-PfCSP IgG_4_ Fab	5.5 (± 0.2) × 10^5^	3.6 (± 0.1) × 10^-3^	6.5 (± 0.1) × 10^-9^
	anti-PfCSP IgM Fab	6.8 (± 0.2) × 10^5^	5.1 (± 0.3) × 10^-3^	7.5 (± 0.2) × 10^-9^
V3 peptide to	anti-gp120 IgA_1_ Fab			1.0 (± 0.1) × 10^-7^
	anti-gp120 IgD Fab			1.0 (± 0.1) × 10^-7^
	anti-gp120 IgE Fab			1.0 (± 0.1) × 10^-7^
	anti-gp120 IgG_1_ Fab			1.1 (± 0.1) × 10^-7^
	anti-gp120 IgG_4_ Fab			1.1 (± 0.1) × 10^-7^
	anti-gp120 IgM Fab			1.1 (± 0.1) × 10^-7^

Data shown are averages from duplicate experiments ± standard deviation (SD).

We then tested binding of the same grass pollen allergen Phl p 7 to a different antibody, expressed as the six isotypes: HAPPIA_1_2, HAPPID2, HAPPIE2, HAPPIG_1_2, HAPPIG_4_2 and HAPPIM2 Fabs (**Supplementary Figure 4**; **Table 2**). Here we observed slightly smaller differences, with affinities ranging ~1.6-fold between 0.8 nM (IgE Fab) and 1.3 nM (IgG_1_ Fab), with ~1.3-fold differences seen in association and dissociation rates. At the highest concentrations of antigen, we observed an induced dissociation phenomenon, where there is a downturn in signal during the association phase, suggesting that the Fab dissociates from anti-His-tag capture during antigen binding. We have previously observed this phenomenon on anti-His-tag chips (16); these artifacts do not substantially affect affinity determination, as induced dissociation data points are excluded from the analysis, and association and dissociation rates are fit separately (**Supplementary Figure 4B**). This induced dissociation phenomenon became more pronounced for the following three Fabs, especially for low molecular weight peptide binders. This artifact may hint at conformational changes upon antigen binding, as discussed later.

To increase the diversity of antibody-antigen binding examined, we assessed ZIKV-EDIII binding to anti-EDIII Fabs (**Supplementary Figure 5**; **Table 2**). The six isotypes exhibited near-identical association rates within a ~1.1-fold range, similar dissociation rates within a ~1.3-fold range, and affinities within a ~1.4-fold range, the highest 5.0 nM (IgE Fab) and lowest 6.9 nM (IgM Fab).

Because SPR measures a change of mass at the surface of the sensor chip (35), low molecular weight peptides are more challenging. Nevertheless, we successfully tested binding of the PfCSP peptide 21 to anti-PfCSP IgA_1_, IgD, IgE, IgG_1_, IgG_4_ and IgM Fabs (**Supplementary Figure 6**; **Table 2**). We observed small differences in the rates, with k_on_ values within a ~1.6-fold range, k_off_ values within a ~1.5-fold range, and the resulting K_D_ values within a ~1.4-fold range from 5.3 nM (IgE Fab) to 7.5 nM (IgM Fab). As the peptide is smaller than 2 kDa, even small amounts of induced dissociation of the ~50 kDa Fab from the anti-His-tag chip will be visually very pronounced. As a result, the k_off_ values were fit to decay to variable instead of being constrained to zero as would otherwise be the case. While we cannot rule out that this large signal-to-noise ratio has a small effect on kinetic and affinity values, there is no ambiguity that the differences in interaction affinities and rates between the isotypes remain small.

Finally, we tested binding of a V3 peptide to the six different anti-gp120 Fab isotypes (**Supplementary Figure 7**; **Table 2**). The binding interaction between peptide and Fabs had fast-on, fast-off kinetics and we therefore performed equilibrium binding analyses. The affinities were nearly identical, with K_D_ values within a ~1.1-fold range. Induced dissociation effects were observed for all isotypes, as described above.

We may conclude from our comparison of five diverse antibody-antigen interactions with six isotypes IgA_1_, IgD, IgE, IgG_1_, IgG_4_ and IgM in Fab format, that only small differences in antigen binding are mediated by the different C_H_1 domains. While these differences in rate constants and affinities (<two-fold) should not be overinterpreted, it is interesting that antibody-antigen interactions for the IgE isotype were consistently stronger (in 4 of the 5 interactions) and had among the fastest association rates (in all four interactions for which k_on_ was determined). Although the sample size is small, it may be the consequence of a yet undefined structural property of the Cϵ1 domain that is transmitted to the antigen-binding site.

### The Fc region affects antigen-binding affinity

**Figure 2:**
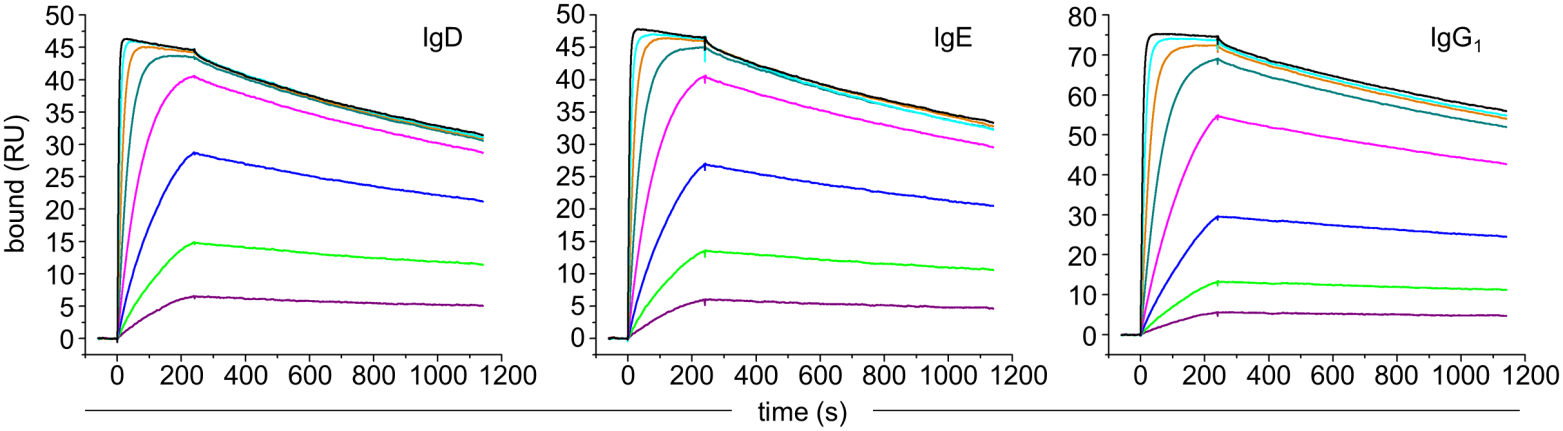
Binding of the antigen Phl p 7 to full-length HAPPI1 antibodies by SPR. HAPPID1, HAPPIE1 and HAPPIG_1_1 were captured on an anti-His-tag chip and the grass pollen allergen Phl p 7 was flowed over in a two-fold dilution series, with the highest concentration 100 nM (black line) and the lowest concentration 1 nM (purple line). Antibodies were captured at slightly different densities. RU, resonance units.

The experiments described so far were performed with Fabs to determine effects mediated by different C_H_1 domains. To assess whether there are differences between Fabs and full-length antibodies of different isotypes, we compared HAPPID1, HAPPIE1 and HAPPIG_1_1, expressed with a His_6_-tag on each of the two heavy chains (**Supplementary Figures 1**, **2B**). As before, we tested binding of a two-fold dilution series of the grass pollen antigen Phl p 7 to antibodies captured on an anti-His-tag chip (**Figure 2**; **Table 3**; **Supplementary Figure 3B**). Differences in antigen-binding kinetics between antibody classes were similar to those seen between Fabs, with a ~1.6-fold difference in association rates, a ~1.3-fold difference in dissociation rates, and a ~1.3-fold difference in affinities. As for the Fabs, IgE displayed the highest affinity and on-rate. Because the bivalent antibodies are captured on the anti-His-tag chip *via* their C-terminus and monomeric antigen is flowed over, the analysis is independent of avidity effects. Surprisingly, binding of the antigen Phl p 7 to full-length antibodies had a ~2.1-fold (IgD), ~1.9-fold (IgE) and ~2.7-fold (IgG_1_) higher affinity compared with their Fab counterparts, implying that the Fc region can allosterically affect antigen binding in a long-range manner, the presence of Fc enhancing antigen-binding affinity.

**Table 3:** Kinetics and affinities of antigen binding to full-length HAPPI1 antibodies.

Antigen	Antibody	k_on_ ± SD (M^-1^ s^-1^)	k_off_ ± SD (s^-1^)	K_D_ ± SD (M)
Phl p 7 to	HAPPID1	2.5 (± 0.1) × 10^6^	3.8 (± 0.2) × 10^-4^	1.6 (± 0.1) × 10^-10^
	HAPPIE1	2.6 (± 0.1) × 10^6^	3.6 (± 0.2) × 10^-4^	1.4 (± 0.1) × 10^-10^
	HAPPIG_1_1	1.6 (± 0.1) × 10^6^	2.9 (± 0.1) × 10^-4^	1.8 (± 0.1) × 10^-10^

Data shown are averages from duplicate experiments ± standard deviation (SD).

### Effects of antigen footprint on long-range Fc-Fab allosteric modulation

**Figure 3:**
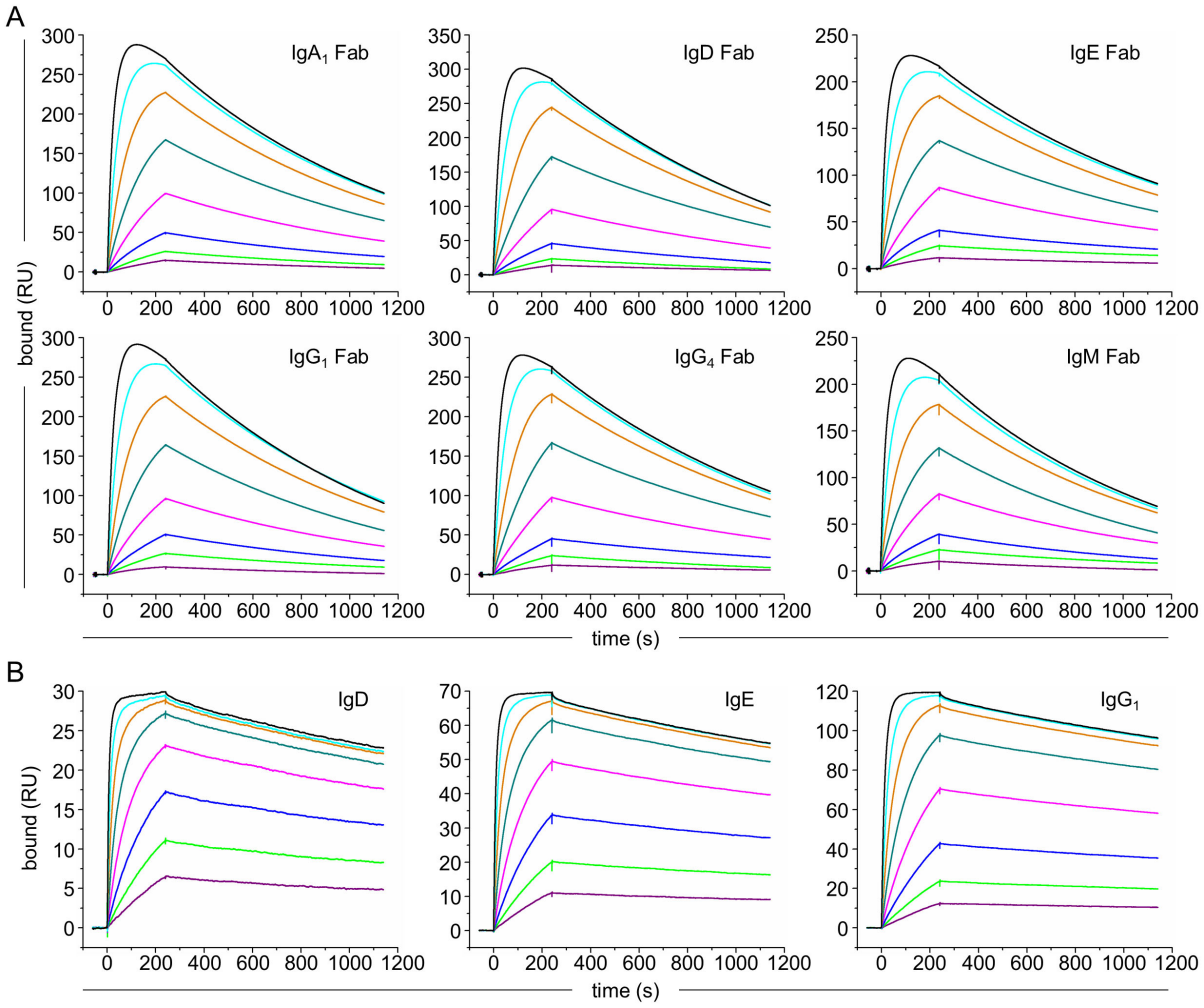
Binding of a paratope-specific Nb to HAPPI1 by SPR. **(A)** HAPPI1 Fabs were captured on an anti-His-tag chip and aδNb072 was flowed over in a two-fold dilution series, with the highest concentration 100 nM (black line) and the lowest concentration 1 nM (purple line). HAPPI1 Fabs were captured at slightly different densities. **(B)** Full-length HAPPI1 antibodies were captured on an anti-His-tag chip and aδNb072 was flowed over in a two-fold dilution series, with the highest concentration 100 nM (black line) and the lowest concentration 1 nM (purple line). Antibodies were captured at different densities. RU, resonance units.

Phl p 7 binds to the HAPPI1 Fab predominantly through the heavy chain, although it also makes contact with two of the three complementarity-determining regions in the light chain (17). To assess whether the nature of the antibody-antigen binding interface affects antibody isotypes differentially, we extended our analysis to a paratope-specific anti-idiotype nanobody (Nb) called aδNb072, raised against HAPPID1, which has the binding characteristics of a native antigen but in comparison with Phl p 7 shows more light chain contact in binding to HAPPI1 Fab (**Supplementary Figure 8**) (16). We measured binding of a two-fold dilution series of aδNb072 to the HAPPIA_1_1, HAPPID1, HAPPIE1, HAPPIG_1_1, HAPPIG_4_1 and HAPPIM1 Fabs captured on an anti-His-tag chip using SPR (**Figure 3A**; **Table 4**; **Supplementary Figure 9A**). aδNb072 bound to the HAPPI1 Fabs with affinities ranging between 2.0 nM and 2.8 nM, a ~1.4-fold difference between the highest affinity, which was yet again for IgE Fab, and the lowest affinity, IgG_1_ and IgD Fab, despite aδNb072 being raised against HAPPID1. We had previously observed a ~2.6-fold difference in affinity when flowing HAPPID1 Fab and HAPPIE1 Fab over aδNb072 captured on an anti-His-tag chip (16), with K_D_ values 1.4 nM and 3.6 nM, respectively, and higher affinity for the IgD Fab. Clearly it matters which binding partner is the immobilized ligand and which is the mobile analyte. For comparison of the binding of antibody isotypes differing solely in their C_H_ sequences, binding of antigen as analyte to immobilized antibody on a sensor surface is likely a more robust method than binding the different antibodies to antigen, being less dependent on different overall electrostatics of the analyte and independent of slight concentration differences.

**Table 4:** Kinetics and affinities of aδNb072 binding to HAPPI1 isotypes.

Nanobody	Antibody	k_on_ ± SD (M^-1^ s^-1^)	k_off_ ± SD (s^-1^)	K_D_ ± SD (M)
aδNb072 to	HAPPIA_1_1 Fab	4.3 (± 0.1) × 10^5^	1.1 (± 0.1) × 10^-3^	2.5 (± 0.1) × 10^-9^
	HAPPID1 Fab	3.8 (± 0.1) × 10^5^	1.1 (± 0.1) × 10^-3^	2.8 (± 0.1) × 10^-9^
	HAPPIE1 Fab	4.6 (± 0.1) × 10^5^	0.9 (± 0.1) × 10^-3^	2.0 (± 0.1) × 10^-9^
	HAPPIG_1_1 Fab	4.0 (± 0.1) × 10^5^	1.1 (± 0.1) × 10^-3^	2.8 (± 0.1) × 10^-9^
	HAPPIG_4_1 Fab	4.1 (± 0.1) × 10^5^	1.0 (± 0.1) × 10^-3^	2.3 (± 0.1) × 10^-9^
	HAPPIM1 Fab	4.4 (± 0.1) × 10^5^	1.2 (± 0.1) × 10^-3^	2.7 (± 0.1) × 10^-9^
aδNb072 to	HAPPID1	1.3 (± 0.1) × 10^6^	2.9 (± 0.1) × 10^-4^	2.3 (± 0.1) × 10^-10^
	HAPPIE1	1.0 (± 0.1) × 10^6^	2.4 (± 0.1) × 10^-4^	2.3 (± 0.1) × 10^-10^
	HAPPIG_1_1	0.7 (± 0.1) × 10^6^	2.1 (± 0.1) × 10^-4^	3.0 (± 0.1) × 10^-10^

Data shown are averages from duplicate experiments ± standard deviation (SD).

Given the differences in binding footprint between the antigen Phl p 7 and the paratope-specific aδNb072, we also assessed binding of a two-fold dilution series of aδNb072 to full-length HAPPID1, HAPPIE1 and HAPPIG_1_1 captured on an anti-His-tag chip (**Figure 3B**; **Table 4**; **Supplementary Figure 9B**). Although the range of k_on_ (~1.9-fold), k_off_ (~1.4-fold) and K_D_ values (~1.3-fold) was comparable to that between the different Fabs, the affinities, ranging from 0.23 nM to 0.30 nM were markedly higher for aδNb072 binding to full-length antibodies compared to the Fabs: ~12.2-fold (IgD), ~8.7-fold (IgE) and ~9.3-fold (IgG_1_) higher affinity, with the largest increase in binding mediated through a slowing of the dissociation rates. These remarkable avidity-independent affinity differences in the presence of the Fc region were much more pronounced for the anti-paratope aδNb072 compared with the antigen Phl p 7. While the electrostatics of a binding surface can impact association rates (36), and the variable regions of a full-length antibody might be expected to be at a greater distance from the negatively-charged CM5 chip than a Fab, dissociation rates would not be expected to be impacted by surface electrostatics in the same way. These results strongly suggest that the Fc region can convey long-range allosteric effects to the V domains in the Fab region, thus impacting binding affinity. The paratope-specific aδNb072 has a more diverse set of interactions across the complementarity-determining regions of both V_H_ and V_L_ domains of HAPPI1 than its antigen Phl p 7 (16), and its binding affinity was more affected by presence or absence of the Fc region. This might suggest that the extent of antigen contact over heavy and light chains influences how an antigen responds to long-range allosteric changes.

### Long-range allosteric effects can be modulated by binders to the Fc region

**Figure 4:**
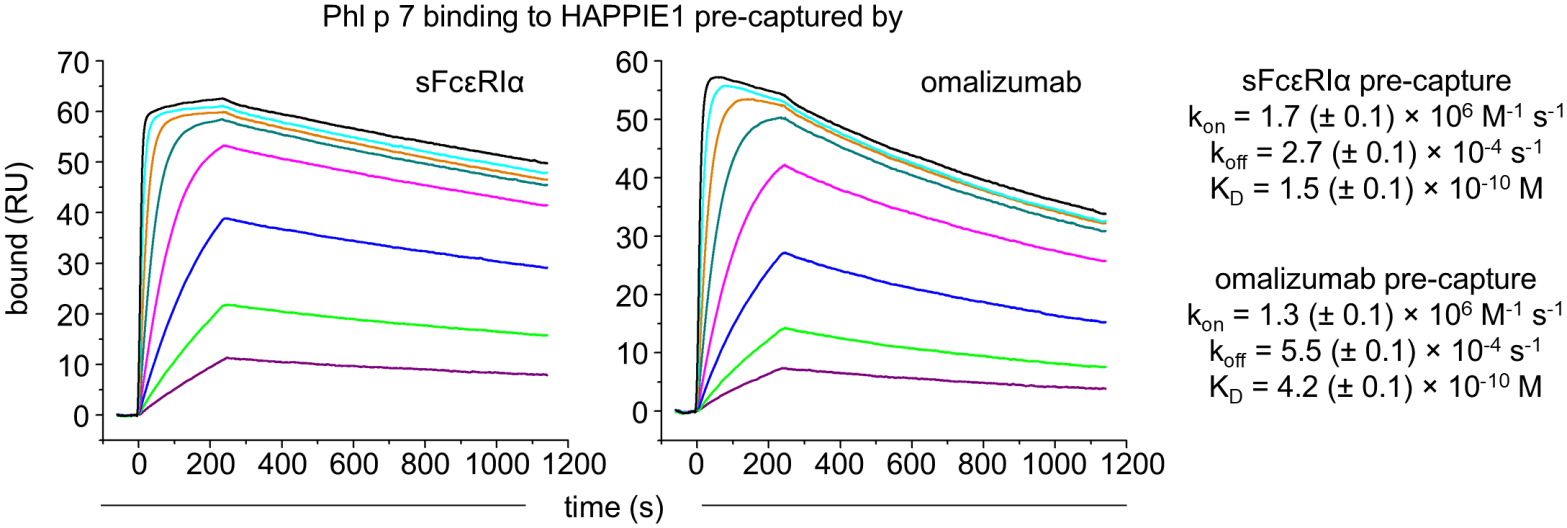
Binding of the antigen Phl p 7 to HAPPIE1 pre-captured via its Fc region. A two-fold dilution series of Phl p 7 was flowed over HAPPIE1 pre-captured by sFcϵRIα or omalizumab Fab, with the highest concentration 100 nM (black line) and the lowest concentration 1 nM (purple line). RU, resonance units.

Given that presence of the Fc region can impact antigen binding, we tested whether the method of antibody capture in SPR could modulate antigen-binding kinetics and affinity. We chose IgE, which is made up of four C_H_ domains without a classical hinge region but with a flexible linker between the Cϵ2 and Cϵ3 domains (37). For capture we used the high-affinity receptor FcϵRI, which binds to the Fc region of IgE and results in IgE-Fc adopting an acutely bent conformation (38), as well as the therapeutic anti-IgE antibody omalizumab (in Fab format), which also binds to the Cϵ3 domain but leads to IgE-Fc adopting a partially bent conformation (29). We captured HAPPIE1 by sFcϵRIα or omalizumab Fab and flowed over a two-fold dilution series of the grass pollen antigen Phl p 7 (**Figure 4**; **Supplementary Figure 10**). There were clear differences between the binding of Phl p 7 to differentially pre-captured HAPPIE1, with a ~2.8-fold difference in affinity observed between receptor- and omalizumab-bound IgE, two ligands known to induce conformational changes in IgE-Fc. These data confirm that long-range allosteric effects are mediated from the Fc region to the Fab region of IgE, which can be modulated by binders to the Fc region.

### Mechanistic insights into long-range allosteric effects within antibodies

To gain mechanistic insights into the long-range allosteric communication within antibodies that modulates antigen binding, we performed thermodynamic analyses. We assessed binding of aδNb072 to the IgG_1_ Fab and full-length IgG_1_ antibody at 8 °C, 16 °C, 24 °C and 32 °C by SPR (**Supplementary Figure 11**) and determined binding kinetics and affinities (**Table 5**). Dissociation at 8 °C showed some biphasic behavior, and here we report only the major dissociation component for both Fab and antibody, which made up over 90% of total binding. As expected, both the association rate and the dissociation rate increased with temperature, but the dissociation rate more markedly, leading to temperature-dependent decreases in affinity, for both Fab and full-length HAPPIG_1_1.

**Table 5:** Temperature-dependence of kinetic data for aδNb072 binding to HAPPIG_1_1.

Interaction	Temperature	k_on_ ± SD (M^-1^ s^-1^)	k_off_ ± SD (s^-1^)	K_D_ ± SD (M)
aδNb072 to HAPPIG_1_1 Fab	8 °C	2.8 (± 0.1) × 10^5^	7.8 (± 0.3) × 10^-5^	2.8 (± 0.1) × 10^-10^
	16 °C	4.6 (± 0.1) × 10^5^	2.3 (± 0.1) × 10^-4^	5.0 (± 0.2) × 10^-10^
	24 °C	7.6 (± 0.1) × 10^5^	8.3 (± 0.1) × 10^-4^	1.1 (± 0.1) × 10^-9^
	32 °C	1.2 (± 0.1) × 10^6^	3.3 (± 0.1) × 10^-3^	2.7 (± 0.1) × 10^-9^
aδNb072 to HAPPIG_1_1	8 °C	3.3 (± 0.1) × 10^5^	4.4 (± 0.1) × 10^-5^	1.3 (± 0.1) × 10^-10^
	16 °C	5.6 (± 0.1) × 10^5^	9.9 (± 0.9) × 10^-5^	1.8 (± 0.2) × 10^-10^
	24 °C	8.8 (± 0.1) × 10^5^	2.3 (± 0.1) × 10^-4^	2.7 (± 0.1) × 10^-10^
	32 °C	1.2 (± 0.1) × 10^6^	4.7 (± 0.1) × 10^-4^	3.8 (± 0.1) × 10^-10^

Data shown are averages from duplicate experiments ± standard deviation (SD).

**Figure 5:**
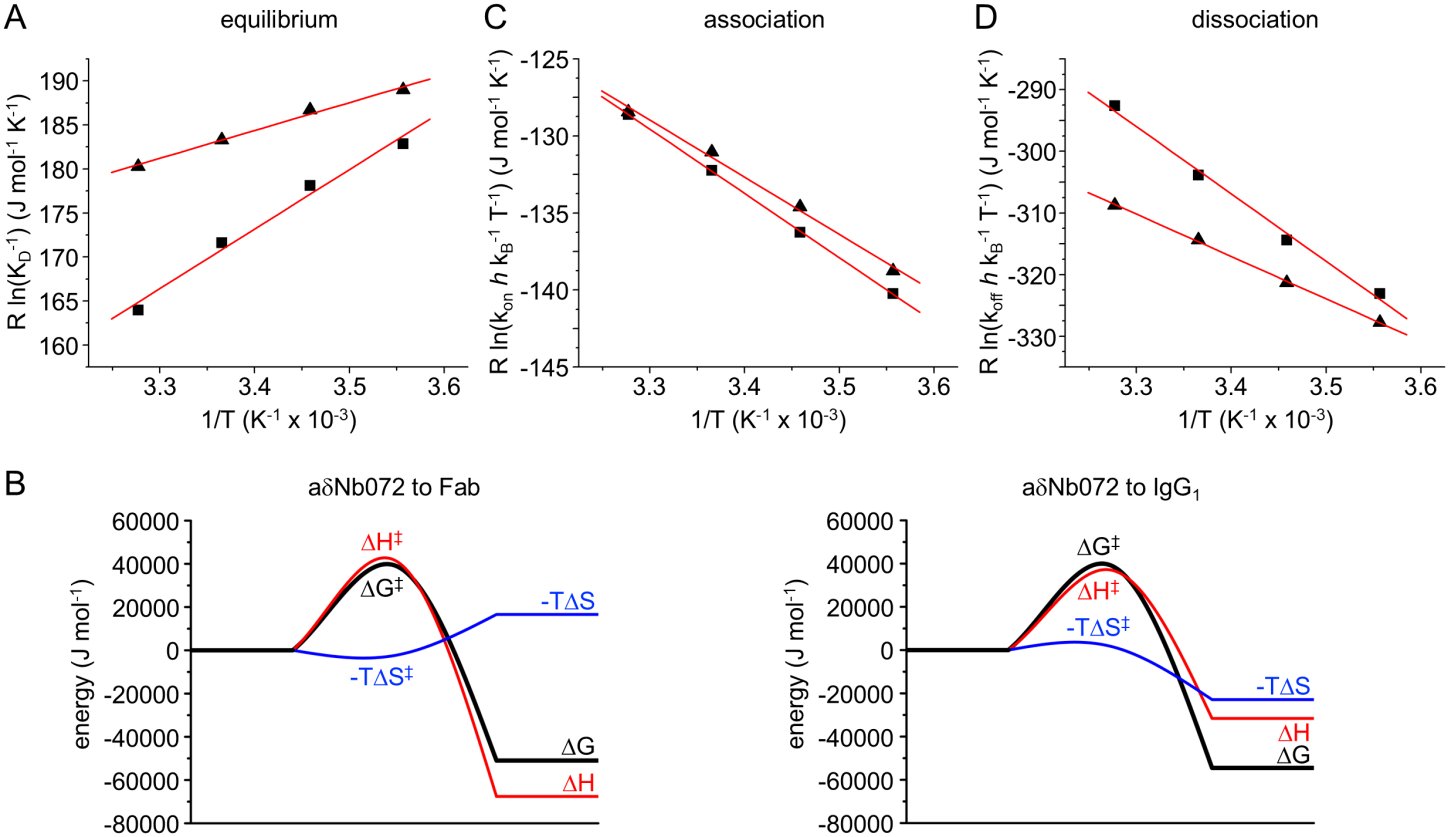
Thermodynamic analysis of binding affinities and rates. **(A)** Van ‘t Hoff plot for equilibrium analysis of the interaction between aδNb072 and HAPPIG_1_1 Fab (squares) or HAPPIG_1_1 (triangles) by SPR at 8 °C, 16 °C, 24 °C and 32 °C, with the fit of the data shown as a red line. **(B)** Reaction profile of aδNb072 binding to IgG_1_ Fab (left panel) or full-length IgG_1_ (right panel) at 24 °C showing enthalpic (red line) and entropic (blue line) contributions to Gibbs free energy (black line). **(C)** Eyring plot of association and **(D)** dissociation rate constants for transition state analysis of the interaction between aδNb072 and HAPPIG_1_1 Fab (squares) or HAPPIG_1_1 (triangles) by SPR at 8 °C, 16 °C, 24 °C and 32 °C, with the fit of the data shown as a red line. R, gas constant; *h*, Planck constant; k_B_, Boltzmann constant.

We performed a van ‘t Hoff analysis to extract the enthalpic and entropic contributions to binding at equilibrium (**Figures 5A, B**, **Supplementary Table 1**). The resulting linear relationship suggests that enthalpy is largely independent of temperature for these interactions (32). Going from the unbound to the bound state, the interaction of aδNb072 with HAPPIG_1_1 Fab was dominated by a favorable enthalpy term but showed an unfavorable entropic component. In contrast, aδNb072’s interaction with full-length HAPPIG_1_1 was considerably less enthalpically favorable, but it had a substantially favorable entropic contribution (**Figure 5B**). Compensation between enthalpy and entropy meant that interaction with both Fab and antibody had similar Gibbs free energy values (**Supplementary Table 1**). These findings suggest that the full-length antibody is pre-organized in a more rigid and less flexible conformation; in binding to aδNb072 fewer favorable interactions are made compared with the Fab but the degrees of freedom – with contributions from both proteins and solvent – increase. Conversely, rigidification of the more flexible Fab in going from the unbound to the bound state results in a decrease in entropy.

To investigate the contribution of the association and dissociation components, we performed an Eyring analysis to determine enthalpies and entropies of activation (**Figures 5B–D**, **Supplementary Table 1**). For association, going from the unbound to the transition state involved a similar large enthalpic barrier for Fab and antibody, with small amounts of entropic gain (Fab) or loss (full-length antibody) (**Figure 5B**). For dissociation, going from the bound to the transition state involved a larger enthalpic barrier for the Fab than the antibody, but whereas the antibody experiences a significant entropic energy barrier during dissociation, for the Fab, entropy *decreases* the total energy barrier for dissociation. These data make it clear that it is the dissociation component that drives the differences observed between Fab and full-length antibody. For a slow dissociation rate, the antigen-binding site appears to require a more rigid environment, which is better provided in the full-length antibody, while in the unconstrained Fab there is more flexibility. In contrast, the association rate is largely unaffected by this. These thermodynamic analyses unambiguously show that the differences in binding of aδNb072 to Fab versus full-length antibody are the result of order being imposed and ensuing entropic penalties.

## Discussion

The effect of changes in antibody isotype (class or subclass), *i.e.*, the C_H_ domains of an antibody, upon its affinity for antigen, determined by the V_H_ and V_L_ domains, has long been a matter for investigation and debate. Does class-switching of an antibody of given antigen specificity have an effect upon antigen affinity? Is there “medium-range” allosteric communication between the C_H_1 domain and the V domains within the Fab region, and/or “long-range” allosteric communication between the C_H_ domains of the Fc and the Fab V domains? These are important questions that require careful comparative analysis to avoid artifacts, such as avidity effects. We therefore generated five sets of antibodies, each set with identical specificities (V_H_ and V_L_ domains) for the given antigen, but for six different isotypes: IgA_1_, IgD, IgE, IgG_1_, IgG_4_ and IgM. These were all produced as Fabs, and for HAPPI1, as IgD, IgE and IgG_1_ full-length antibodies. We therefore had the tools to investigate intra-Fab modulation of antigen affinity by changing the C_H_1 domain, for a range of different antigens, and Fc-Fab communication, also for two different antigenic “footprints”, namely the native antigen and a paratope-directed nanobody.

Comparing the antigen binding between IgA_1_, IgD, IgE, IgG_1_, IgG_4_ and IgM Fabs, we observed small, less than two-fold differences in binding kinetics and affinities. Differences between isotypes were also similarly small when comparing binding of antigen to full-length IgD, IgE and IgG_1_ for one of the antibody-antigen interactions. We chose a range of diverse antigens for our analysis, both proteins and peptides, that contact heavy and light chain to different extents (17, 19, 22, 34). The corresponding antibodies contained both frequently as well as less frequently occurring V_H_ and V_L_ domain families (39), with representation of both λ and κ light chains, and diversity in V_L_ domain family use (V_λ_1, V_κ_1, V_κ_4 and V_λ_3). A comparison of binding of the grass pollen allergen Phl p 7 to the HAPPIA1 and HAPPIG1 subclasses pre-captured via their λ light chains has previously been performed, with small differences reported (8). We extended this analysis for Fabs to all classes of antibodies by including IgD, IgE and IgM, but did not observe any greater differences. Small differences in antigen-binding affinity and kinetics, independent of antibody avidity effects, have previously been reported by other groups. Comparing IgA_2_ and IgG_1_ isotypes, a 3-fold and 1.4-fold difference in antigen-binding affinity has been reported for two anti-HIV-1 antibodies (7), of V_H_3 and V_λ_2, and V_H_1 and V_κ_1 family, respectively (40). It should be noted that a comparison was made between IgA_2_ directly immobilized onto a sensor chip by amine-coupling and IgG_1_ reversibly captured by anti-Fc (7), which could add some complexity to direct comparisons between isotypes. Subtle avidity-independent differences between the nine human antibody classes and subclasses for Trastuzumab and Pertuzumab, both of V_H_3 and V_κ_1 family, as well as for different light chain constant domains, have also previously been observed (9). Reports of large affinity differences, between IgA_1_ and IgG_1_ Fabs in binding to tubulin, of V_H_3 and V_κ_2 family (10), or between IgA_1_ and IgG_1_ Fabs in binding to HIV-1 glycoproteins (11), of V_H_3 and V_λ_1 or V_H_6 and V_κ_1 family (41), may need to be interpreted with caution. It should be noted that some studies make assessing the quality of differences reported between antibody isotypes difficult, by not including a visual representation of the underlying data, while in other studies, visualization of SPR data informs the expert reader of weaknesses in experimental design, analysis performed and/or conclusions drawn. Given our comprehensive analysis, as well as the results published by other groups, we can conclude that small, less than 5-fold differences in antigen-binding affinity can be caused by changing the C_H_ domains in most antibodies. However, it is possible that certain V_H_ and V_L_ families, as well as certain combinations of V_H_ and V_L_ domains are more amenable to such isotype-mediated allosteric effects on antigen binding. The antigen footprint, *i.e.*, the extent and nature of contacts that the antigen makes with the V_H_ and V_L_ domains, could affect the susceptibility to allosteric conformational changes in the antigen-binding site. Peptide antigens may occupy less surface area than protein antigens (42), but in the results reported in this study we see no obvious differences between proteins and peptides, although we did not study any very large antigens. Magnitude of antigen-binding affinity and kinetic rates may also affect susceptibility; here we saw a small reduction in differences among the Fabs as antigen-binding affinity decreased, with almost no differences seen for the fast-on, fast-off kinetic curves of V3 peptide binding to anti-gp120 Fabs.

Having a monomeric antigen flow over antibodies removes avidity-dependent binding effects, as well as differences arising from overall antibody electrostatics and small differences in concentration, allowing a clean comparison of C_H_ domain-mediated differences. Use of an anti-His-tag capture of the Fabs and antibodies avoids artifacts resulting from covalent immobilization of diverse antibody isotypes and differential behavioral changes upon regeneration. However, anti-His-tag capture brought with it its own set of problems for some of the Fab-antigen pairs. We can only speculate as to the cause of the induced dissociation behavior we observed at higher concentrations of antigen, with the Fabs only carrying one His_6_-tag in contrast to the full-length antibodies. This phenomenon was very pronounced for binding of peptides, where the Fab has a more than 20-fold higher molecular weight, and loss of very small amounts of captured Fab would have a large visual effect on binding of antigenic peptide. Given the differences we have seen in antigen binding as a result of long-range allosteric mechanisms, it is possible that the induced dissociation observed could be the result of an allosteric conformational change within the Fab, leading to release from the anti-His-tag capture antibody.

We observed larger avidity-independent differences between Fabs and their full-length counterparts than we did between the different isotypes. The antigen Phl p 7 bound with two- to three-fold higher affinity to full-length antibodies, while the anti-paratope Nb showed as much as ten-fold higher affinity. The presence of the Fc region allosterically enhanced antigen binding in a manner sensitive to the nature of the paratope’s footprint, particularly the extent of contact across the heavy and light chains, suggesting long-range communication between these distant regions. We have provided evidence of a clear entropy-driven mechanism: binding of the Nb to IgG_1_ Fab was entropically unfavorable, whereas binding to full-length IgG_1_ was entropically favorable, resulting in a slower dissociation rate for binding to full-length IgG_1_. These data suggest that the full-length antibody is more rigidly pre-organized for antigen binding, leading to a higher binding affinity, than the more flexible Fab. While we observed affinity differences between HAPPID1 Fab and full-length HAPPID1 as a result of allosteric communication between Fc and Fab, differential scanning calorimetry of HAPPID1 Fab, full-length HAPPID1 and IgD-Fc shows that the Fc region does not affect thermostability of the Fab region (43). Greater than 6-fold and 16-fold lower affinities of two peptides of different lengths binding to an anti-HIV-1 Fab compared with its full-length IgG have been reported using ITC, with similar favorable enthalpy values for binding, and the full-length antibody less entropically unfavorable than the Fab (44). On the other hand, only subtle affinity differences have been shown for a mouse Fab and its antibody, with the Fab more entropically unfavorable in some conditions, but not all (45). Notably, IgE exhibited the highest affinity in Fab format and the smallest affinities shift upon transition to the full-length antibody. While structural data for the Cε1 domain are limited, our thermodynamic results suggest that the IgE antigen-binding site may be more functionally pre-organized than other isotypes, possibly due to the unique conformational constraints inherent to the IgE format.

Allosteric communication within the Fc region of IgE has been well studied, with FcϵRIα and omalizumab binding imparting different degrees of bending on IgE-Fc (29, 38). We observed a ~2.8-fold difference in antigen-binding affinity as a result of pre-capture of IgE by FcϵRIα or omalizumab. These results confirm that differences in the Fc region of IgE are also conveyed into the Fab region and the antigen-binding site, with the partially bent conformation of IgE-Fc possibly resulting in a less rigid antibody molecule. Allosteric communication from the Fab to the Fc region has previously been reported in IgE, with V_H_ sequence suggested to modulate binding of IgE to FcϵRIα, but only subtle differences were seen in binding to omalizumab (46). For other antibody isotypes such as IgG there have been reports of antigen binding affecting receptor binding sites (14, 47) or the protein A binding site (48) in the Fc region. It is currently not understood how allosteric changes, including rigidification, could be communicated across a flexible hinge region from Fc to Fab (and *vice versa*), and hinge flexibility varies considerably between antibody classes and subclasses. Unlike the IgA, IgD, and IgG isotypes which utilize a hinge, the IgE isotype contains an additional C_H_ domain pair in its place, connected to the rest of the Fc region via a flexible linker (37). It remains unknown whether the structured IgE format is more or less amenable to mediating conformational changes than the unstructured hinge configuration.

Conformational changes upon antigen binding, propagated within membrane-bound antibodies, or B cell receptors (BCRs), are a key feature of one of the main four proposed models for BCR triggering, so-called “conformation-induced oligomerization”, and it remains to be determined whether and how a monovalent antigen could achieve this (49). Our data suggest that antigen binding to the V domains can sense the presence or absence of the Fc region, *via* long-range allosteric effects that involve antibody rigidity/flexibility. In a BCR, the reverse might be true with the presence of even monovalent antigen being sensed in this way. Structural data are still lacking to support bi-directional long-range allosteric effects within antibodies, and further studies will be necessary to fully understand information transfer between Fab and Fc, in both soluble and membrane-bound antibodies.

How can we harness knowledge on affinity modulation from antibody format, isotype and binders to the Fc region? The small, less than two-fold affinity differences observed between isotypes would likely provide only limited physiological benefits, while the larger three- to ten-fold differences imparted by the Fc region might have substantial effects on antibody occupancy, depending on biological concentrations: a ten-fold reduction in affinity could mean the difference between 50% and ~9% of antibodies being bound to antigen (**Supplementary Figure 12**). Assessing affinity in Fab format or using antibody pre-capture in SPR experiments are both standard approaches for determining the affinity of antibodies in an avidity-independent manner. The current study suggests that both approaches may be susceptible to long-range allosteric modulation, and integrating an understanding of these allosteric effects may improve workflows in the design and optimization of antibody therapeutics. Diagnostic applications such as lateral flow tests may benefit from even small improvements in association rate and affinity in the detection of low abundant antigens, and exploring IgE as an alternative to the conventional IgG could be impactful. Our findings on antigen-binding site pre-organization and rigidity could lend themselves to antibody stability engineering for affinity enhancement, as well as affinity modulation of native antibodies through binders to the Fc region.

## Data Availability

The raw data supporting the conclusions of this article will be made available by the authors, without undue reservation, to any qualified researcher.
