# Supplementary material for: Long-range allosteric communication within antibodies affects antigen-binding affinity
Source: Front Immunol. 2026 Jul 3;17:1865402. doi: 10.3389/fimmu.2026.1865402 (PMC13375461; doi:10.3389/fimmu.2026.1865402)
Supplement: Supplementary file 1 [file Image1.pdf]

A heavy chain

V_H

```
HAPPI1      QVQLVQSGAEVRNPGASVKVSCKASGYTF------TSYAIHWVRQAPGHRLEWVGRINTDNGNTKYSQKFHGRVALSRDTSASTTYMDLSSLNSEDTAVYYCARAFYY-------SSGVMFDSWGQGALVTVSS
HAPPI2      QVQLQESGPGLVKPSETLSLTCTVSGGSINS----SSYFWGWIRQPPGKGLEWIGSIYY-SGSTHYSPSLKSRVTISVDTSKSQFSLKLRSVTAADTAVYFCARRRWWEQL------GGAVDIWGQGTMVTVSS
anti-EDIII  EVQLVQSGAEVKKPGASVKVSCKASGYTFTSGHTFPSYDINWVRQATGQGLEWMGWMNPNRGNTGYAQKFQGRVTMTRNTSINTAYMELSSLRSEDTAVYYCARVRSGTNYGSYYYYYGMDVWGQGTTVTVSS
anti-PfCSP  QVQLVQSGAEVKKPGASVKVSCKASGYTF------TSYAIHWVRQAPGQRLEWMGWIKAGNGNTRYSQKFQDRVTITRDTSTTTAYMELSSLRSEDTAVYYCALLTVL-------TPDDAFDIWGQGTMVTVSS
anti-gp120  QVQLVQSGAEVKKPGASVKVSCKASGYTF------TNYAIHWVRQAPGHRLEWMGWINGGDGNTKYSQKLQGRVTITRDTSASTAYMELSSLRSEDSAVYYCMRAYYY---GSRGLVDDALDVWGQGTLVTVSS
```

Fabs and full-length: C_H1

```
IgA1  ASPTSPKVFPLSLCSTQP---DGNVVIACLVQGFFPQEPLSVTWSESGQG--VTARNFPPSQDASGDLYTTSSQLTLPATQCLAG--KSVTCHVKHYTNP-SQDVTVPCPVPS-
IgD   APTKAPDVFPIISGCRHPK-DNSPVVLACLITGYHP-TSVTVTWYMGTQS--QPQRTFPEI-QRRDSYYMTSSQLSTPLQQWRQ---GEYKCVVQHTASKSKKEIFRWPESPKA
IgE   ASTQSPSVFPLTRCCKNIPSNATSVTLGCLATGYFP-EPVMVTWDTGSLN--GTTMTLPATTLTLSGHYATISLLTV-SGAWAK---QMFTCRVAHTPSSTDWVDNKTFSVCS-
IgG1  ASTKGPSVFPLAPSSKSTS--GGTAALGCLVKDYFP-EPVTVSWNSGALT--SGVHTFPAV-LQSSGLYSLSSVVTVPSSSLGT---QTYICNVNHKPSNTKVDKKVEPKSCD-
IgG4  ASTKGPSVFPLAPCSRSTS--ESTAALGCLVKDYFP-EPVTVSWNSGALT--SGVHTFPAV-LQSSGLYSLSSVVTVPSSSLGT---KTYTCNVDHKPSNTKVDKRVESKYGP-
IgM   GSASAPTLFPLVSCENSPS-DTSSVAVGCLAQDFLP-DSITFSWKYKNNSDISSTRGFPSV--LRGGKYAATSQVLLPSKDVMQGTDEHVVCKVQHPNG--NKEKNVPLPVIA-
```

full-length only

```
IgD   QASSVPTAQPQAEGSLAKATTAPATTRNTGRGGEEKKKEKEKEEQEERETKTPECPSHTQPLGVYLLTPAVQDLWLRDKATFTCFVVGSDLKDAHLTWEVAGKVPTGGVEEGLLERHSNGSQSQHSRLTLPRSLWNAGTSVTCTLNHPSLPPQRLMALREP
      AAQAPVKLSLNLLASSDPPEAASWLLCEVSGFSPPNILLMWLEDQREVNTSGFAPARPPPQPRSTTFWAWSVLRVPAPPSPQPATYTCVVSHEDSRTLLNASRSLEVSYVTDHGPMK

IgE   RDFTPPTVKILQSSCDGGGHFPPTIQLLCLVSGYTPGTINITWLEDGQVMDVDLSTASTTQEGELASTQSELTLSQKHWLSDRTYTCQVTYQGHTFEDSTKKCADSNPRGVSAYLSRPSPFDLFIRKSPTITCLVVDLAPSKGTVNLTWSRASGKPVNHST
      RKEEKQRNGTLTVTSTLPVGTRDWIEGETYQCRVTHPHLPRALMRSTTKTSGPRAAPEVYAFATPEWPGSRDKRTLACLIQNFMPEDISVQWLHNEVQLPDARHSTTQPRKTKGSGFFVFSRLEVTRAEWEQKDEFICRAVHEAASPSQTVQRAVSVNPGK

IgG1  KTHTCPPCPAPELLGGPSVFLFPPKPKDTLMISRTPEVTCVVVDVSHEDPEVKFNWYVDGVEVHNAKTKPREEQYNSTYRVVSVLTVLHQDWLNGKEYKCKVSNKALPAPIEKTISKAKGQPREPQVYTLPPSRDELTKNQVSLTCLVKGFYPSDIAVEWE
      SNGQPENNYKTTPPVLDSDGSFFLYSKLTVDKSRWQQGNVFSCSVMHEALHNHYTQKSLSLSPGK
```

tag

```
tag   GGGGSHHHHHH
```

B light chain

V_L

```
HAPPI1      QSALTQPPSVSGAPGQRVSISCTGGSSNFGAGYDVHWYQQLPATAPKLLIYGNNNRPSGVPDRFSGSKSGTSASLAITGLQAEDEGDYFCQSFDTSLSG-WIFGGGTKLTVL
anti-EDIII  SYELTQPPSASGTPGQRVTISCSGSSSNIGNNY-VHWYQQLPGSAPKLLIYRNNQRPSGVPDRFSGSKSGTSGSLAISGLRSEDEADYYCASWDDSLSGHWVFGGGTKVTVL
anti-gp120  SYELTQPPSVSVSPGQTARITCSGDELPKKY---AYWYQEKSGQAPVLIIYEDSKRPSGIPERFSGSSSGTMATLTISGAQVEDEADYYCFSTDSS-GDLWVFGGGTKLTVL
```

C_L

```
λ2          GQPKAAPSVTLFPPSSEELQANKATLVCLISDFYPGAVTVAWKADSSPVKAGVETTTPSKQSNNKYAASSYLSLTPEQWKSHRSYSCQVTHEGSTVEKTVAPTECS
```

C light chain

V_L

```
HAPPI2      AIQMTQSPSSLSASVGDRVTITCRASQDI------RNDLGWYQQKPGKAPKLLIYAASSLQSGVPSRFSGSGSGTDFTLTISSLQPEDFATYYCLQDYNYVALTFGGGTKVEIK
anti-PfCSP  DIVMTQSPDSLAVSLGERATINCKSSQSVLYSSNNKNYLAWYQQKPGQPPNLLIYWASTRQSGVPDRFSGSGSGTDFTLTISSLQAEDVAVYYCHQYYS-SPLTFGGGTKVEIK
```

C_L

```
κ           RTVAAPSVFIFPPSDEQLKSGTASVVCLLNNFYPREAKVQWKVDNALQSGNSQESVTEQDSKDSTYSLSSTLTLSKADYEKHKVYACEVTHQGLSSPVTKSFNRGEC
```

Figure S1: Sequences of antibodies used in this study. (A) Sequences of the heavy chains of Fabs and antibodies, from the N-terminus to the C-terminal His_6-tag. Variable (V_H) and constant (C_H1) heavy chain domains were aligned with Clustal Omega. (B) Variable (V_L) and constant (C_L) light chain domain sequences of the λ light chain Fabs. V_L domains were aligned with Clustal Omega. (C) V_L and C_L sequences of the κ light chain Fabs. V_L domains were aligned with Clustal Omega. Approximate locations of complementarity-determining regions are colored in blue.
