# Supplementary figures and images for: Long-range allosteric communication within antibodies affects antigen-binding affinity
Source: Front Immunol. 2026 Jul 3;17:1865402. doi: 10.3389/fimmu.2026.1865402 (PMC13375461; doi:10.3389/fimmu.2026.1865402)

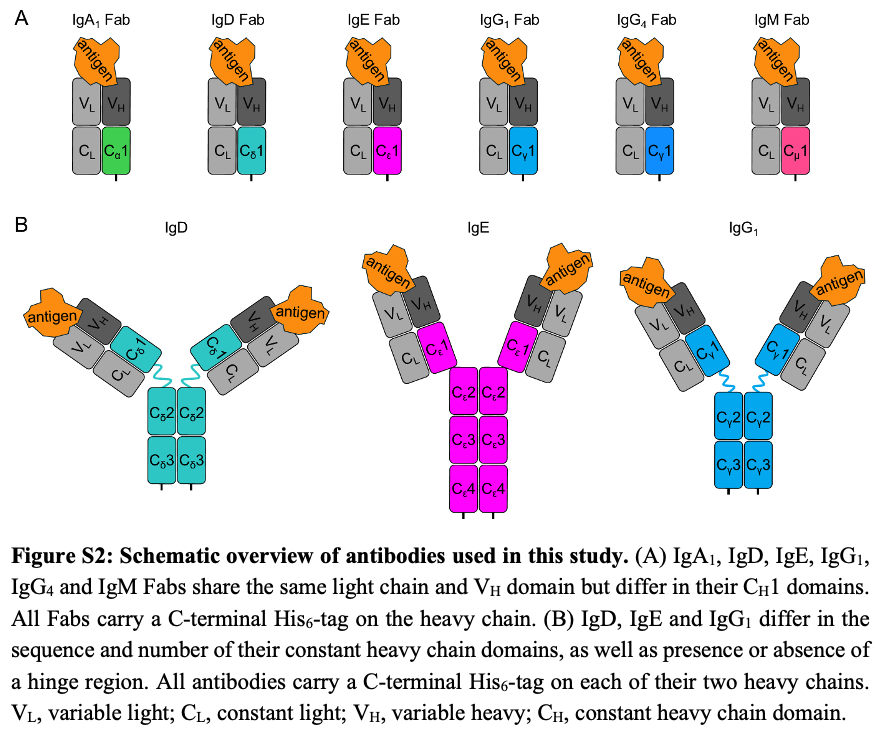

Supplement: Supplementary file 2 [file Image2.png]

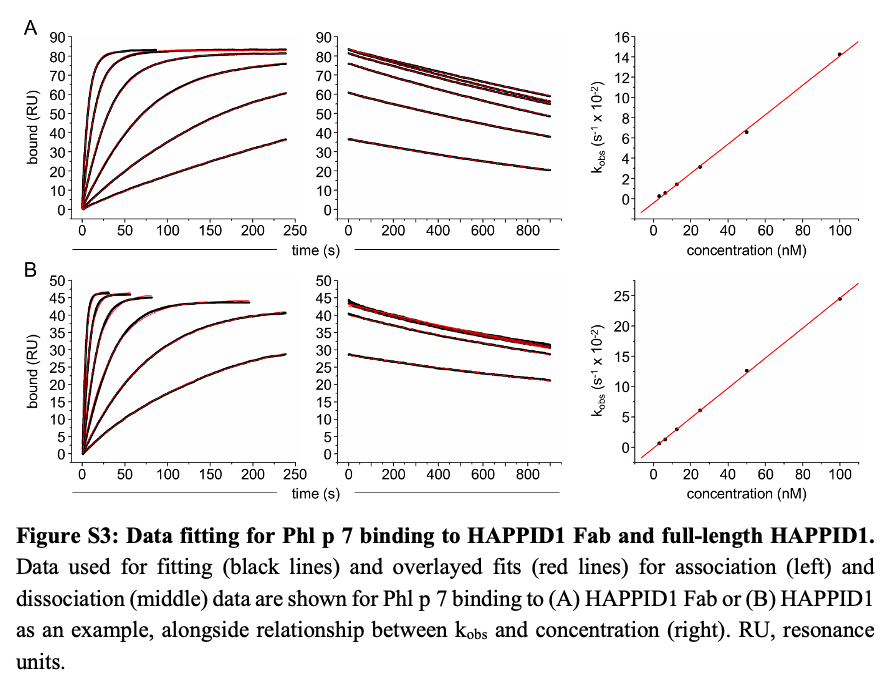

Supplement: Supplementary file 3 [file Image3.png]

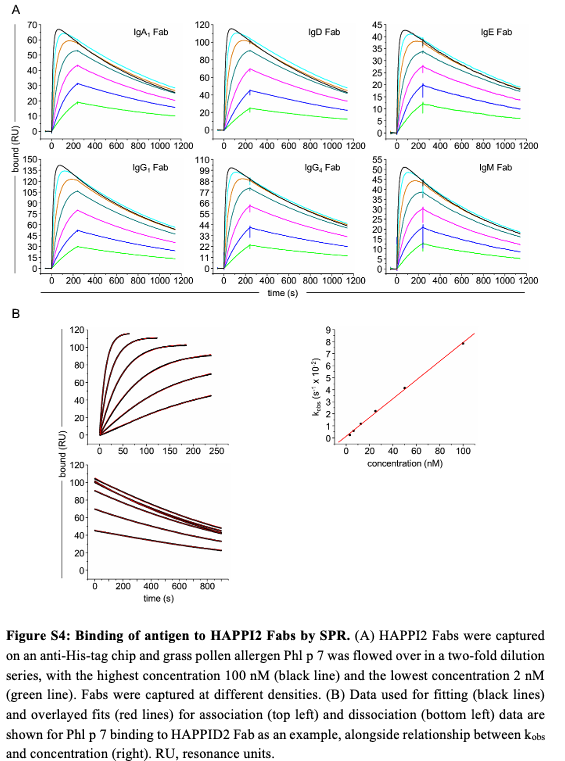

Supplement: Supplementary file 4 [file Image4.png]

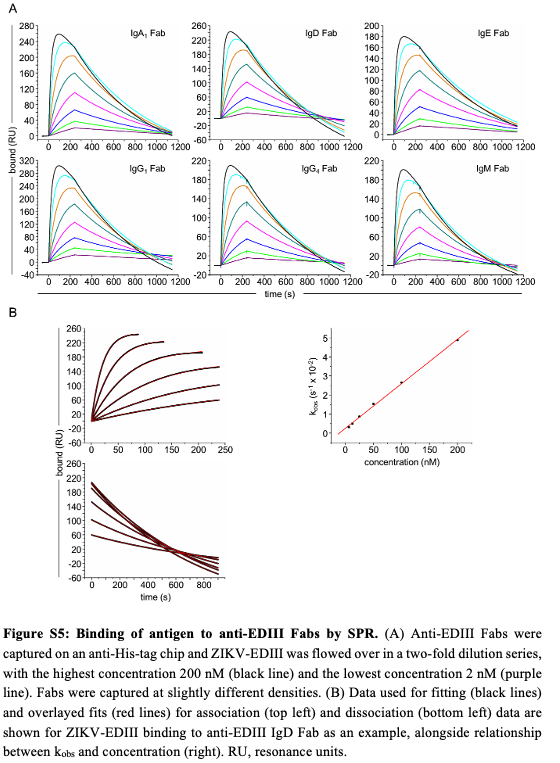

Supplement: Supplementary file 5 [file Image5.png]

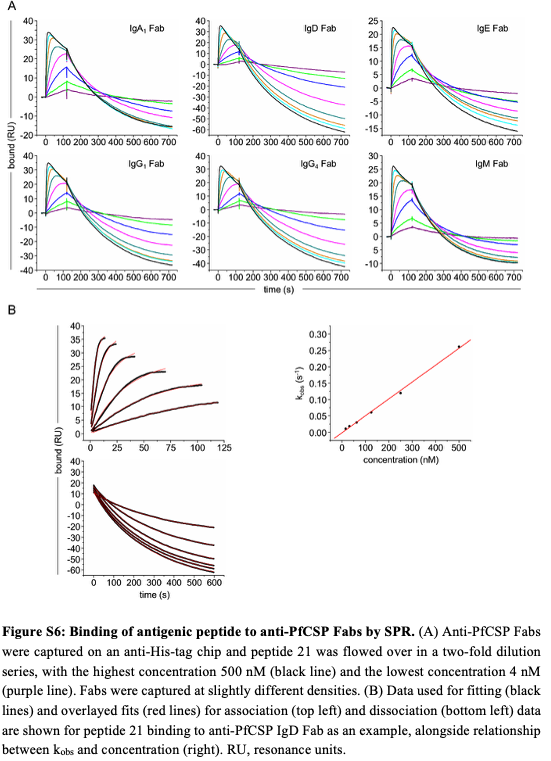

Supplement: Supplementary file 6 [file Image6.png]

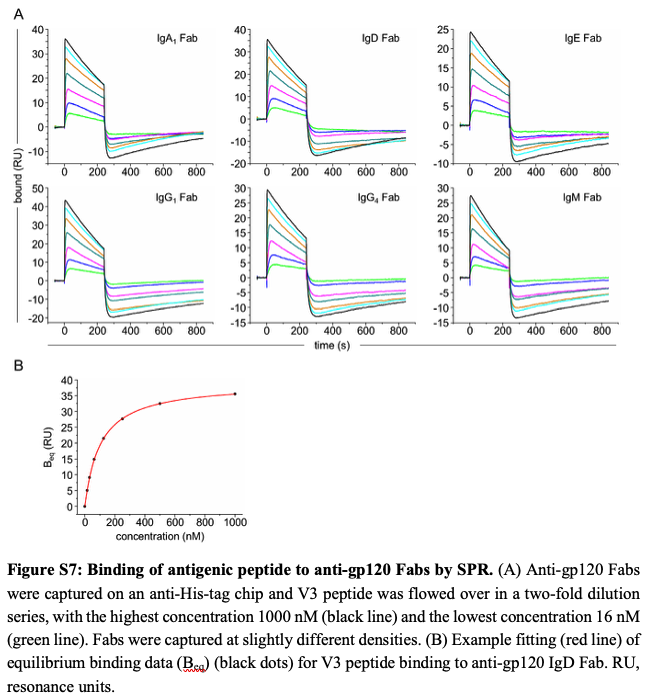

Supplement: Supplementary file 7 [file Image7.png]

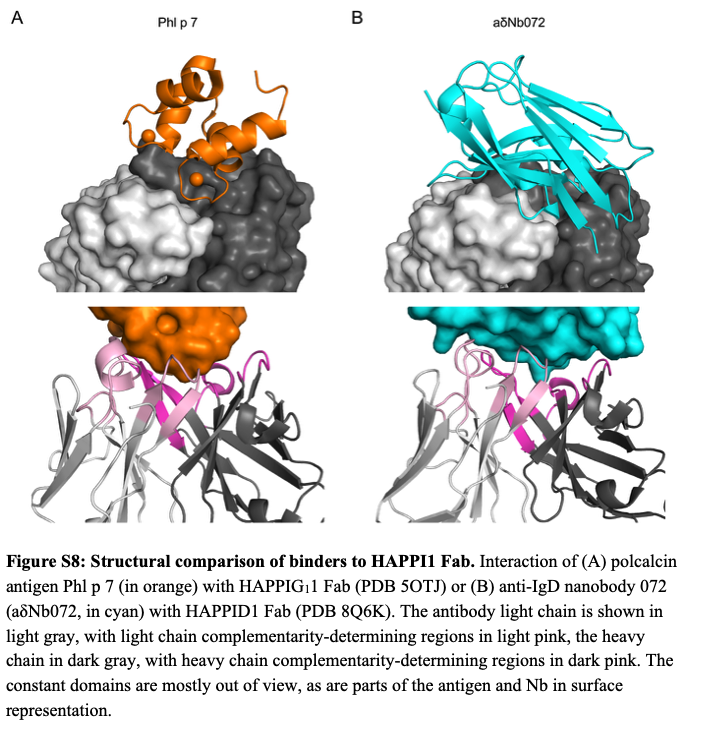

Supplement: Supplementary file 8 [file Image8.png]

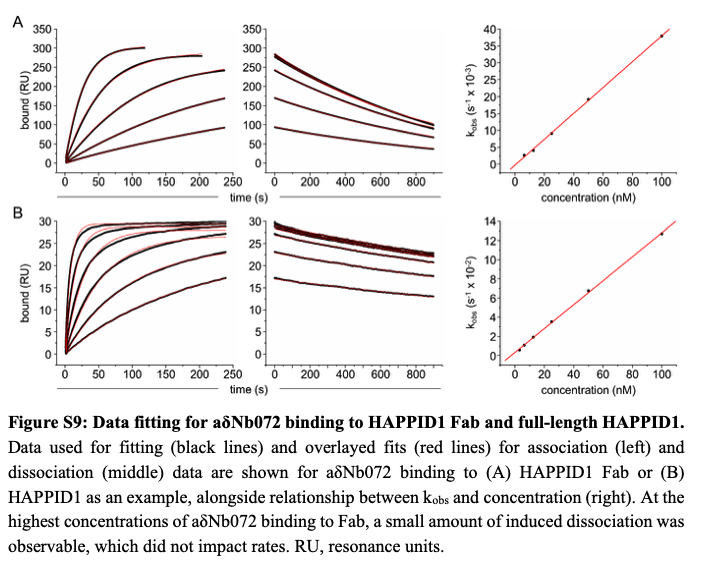

Supplement: Supplementary file 9 [file Image9.png]

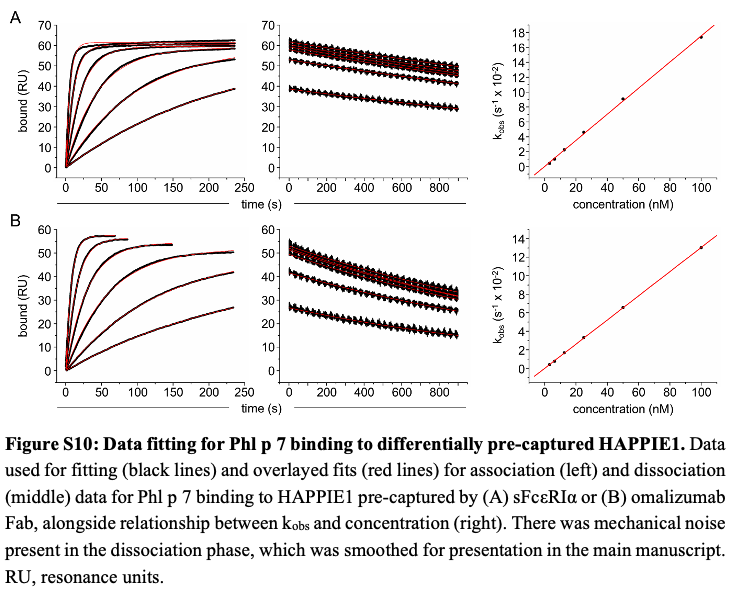

Supplement: Supplementary file 10 [file Image10.png]

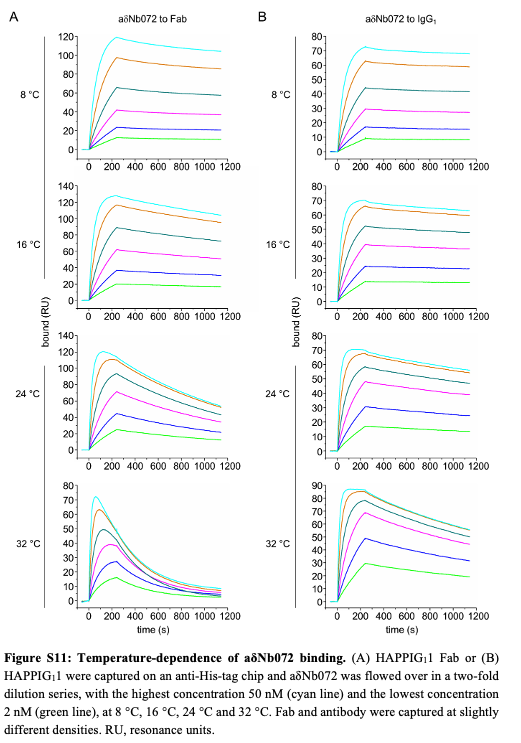

Supplement: Supplementary file 11 [file Image11.png]

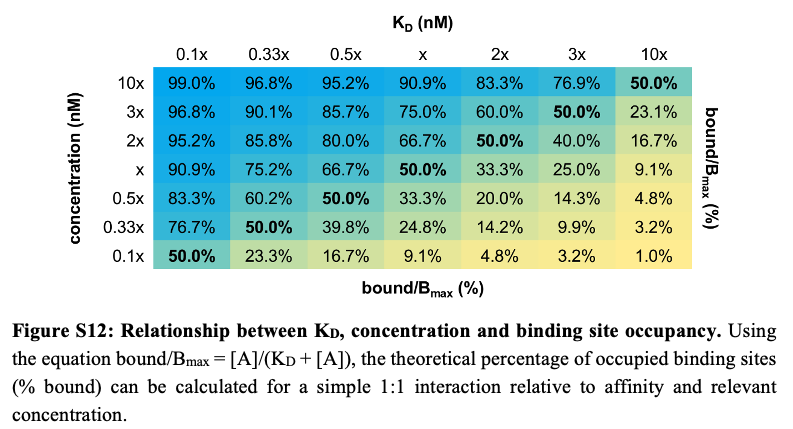

Supplement: Supplementary file 12 [file Image12.png]

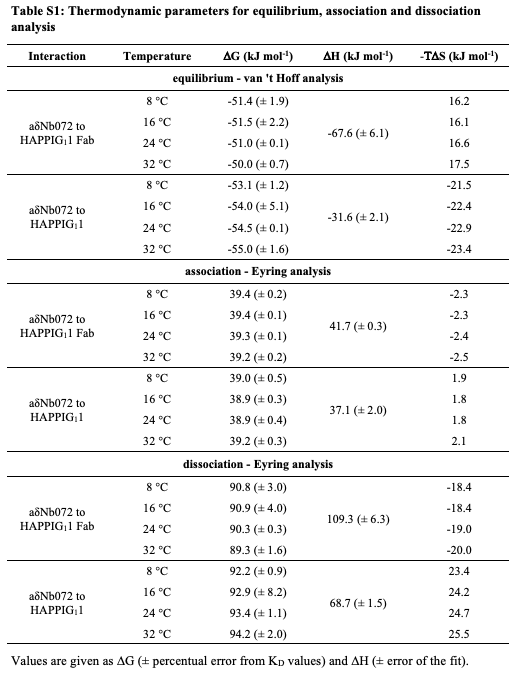

Supplement: Supplementary file 13 [file Supplementaryfile1.png]
